# Understanding collective suicides in Morocco: A 35-year epidemiological study

**DOI:** 10.1192/j.eurpsy.2024.1650

**Published:** 2024-08-27

**Authors:** S. Boukhorb, S. Hmimou, S. Irnat, F. Hadrya, N. Rhalem, M. A. Bellimam, A. Soulaymani, A. Mokhtari, R. Soulaymani-Bencheikh, H. Hami

**Affiliations:** ^1^Laboratory of Biology and Health, Faculty of Sciences, Ibn Tofail University, Kenitra; ^2^University Hassan First of Settat, Higher Institute of Health Sciences, Health Sciences and Technologies Laboratory, Settat; ^3^ Moroccan Poison Control Center; ^4^Forensic Institute of Royal Gendarmerie, Rabat, Morocco

## Abstract

**Introduction:**

Suicide is a major public health concern, ranking among the leading causes of death worldwide.

**Objectives:**

This study investigated the epidemiological features of collective suicide incidents in Morocco.

**Methods:**

We performed a retrospective analysis of suicidal poisoning cases recorded by the Moroccan Poison Control Center (MPCC) over a 35-year period.

**Results:**

During the study’s duration, the MPCC recorded 168 suicide cases. The mean age of those involved in these incidents was 23.9 years, with a female-to-male ratio of 1.57. The majority of those affected were adolescents and young adults, specifically between 15 and 34 years of age. Pesticides and drugs were the most commonly used methods of suicide and accounted for 31.1% and 20.1% of the cases, respectively. The majority of incidents occurred in the home environment and were primarily caused by oral exposure. The symptoms of poisoning varied according to the consumed substance, amount ingested, and elapsed time until medical treatment. The symptoms included disturbances in the neurological, gastrointestinal, respiratory, and cardiovascular systems. Of the 100 cases with known outcomes, one person died due to poisoning, whereas the remaining cases survived, although some enduring complications.

**Conclusions:**

Suicide accounted for 1.3% of global mortality, ranking as the 17th most common cause of death in 2019, according to data from the World Health Organization (WHO). This trend emphasizes the urgent need for continuous efforts at all levels to address and mitigate this rapidly growing issue.

**Disclosure of Interest:**

None Declared

